# The highly accurate anteriolateral portal for injecting the knee

**DOI:** 10.1186/1758-2555-3-6

**Published:** 2011-03-30

**Authors:** Colbert E Chavez-Chiang, Wilmer L Sibbitt, Philip A Band, Natalia R Chavez-Chiang, Suzanne L DeLea, Arthur D Bankhurst

**Affiliations:** 1 Department of Internal Medicine, University of New Mexico Health Sciences Center, Albuquerque, NM, USA; 2 the Departments of Orthopaedic Surgery and Pharmacology, New York University Medical Center, New York, NY, USA

## Abstract

**Background:**

The extended knee lateral midpatellar portal for intraarticular injection of the knee is accurate but is not practical for all patients. We hypothesized that a modified anteriolateral portal where the synovial membrane of the medial femoral condyle is the target would be highly accurate and effective for intraarticular injection of the knee.

**Methods:**

83 subjects with non-effusive osteoarthritis of the knee were randomized to intraarticular injection using the modified anteriolateral bent knee versus the standard lateral midpatellar portal. After hydrodissection of the synovial membrane with lidocaine using a mechanical syringe (reciprocating procedure device), 80 mg of triamcinolone acetonide were injected into the knee with a 2.0-in (5.1-cm) 21-gauge needle. Baseline pain, procedural pain, and pain at outcome (2 weeks and 6 months) were determined with the 10 cm Visual Analogue Pain Score (VAS). The accuracy of needle placement was determined by sonographic imaging.

**Results:**

The lateral midpatellar and anteriolateral portals resulted in equivalent clinical outcomes including procedural pain (VAS midpatellar: 4.6 ± 3.1 cm; anteriolateral: 4.8 ± 3.2 cm; p = 0.77), pain at outcome (VAS midpatellar: 2.6 ± 2.8 cm; anteriolateral: 1.7 ± 2.3 cm; p = 0.11), responders (midpatellar: 45%; anteriolateral: 56%; p = 0.33), duration of therapeutic effect (midpatellar: 3.9 ± 2.4 months; anteriolateral: 4.1 ± 2.2 months; p = 0.69), and time to next procedure (midpatellar: 7.3 ± 3.3 months; anteriolateral: 7.7 ± 3.7 months; p = 0.71). The anteriolateral portal was 97% accurate by real-time ultrasound imaging.

**Conclusion:**

The modified anteriolateral bent knee portal is an effective, accurate, and equivalent alternative to the standard lateral midpatellar portal for intraarticular injection of the knee.

**Trial Registration:**

ClinicalTrials.gov: NCT00651625

## Background

Intraarticular injection of the knee is the most common invasive procedure in sports medicine, accounting for approximately 39-64% of all outpatient joint procedures [1-10]. A number of anatomic landmark palpation-guided intraarticular injection approaches to the knee are used including the extended leg lateral or medial midpatellar approaches and the bent knee lateral and medial anterior approaches [4,6,10-18]. While accessing the anteriolateral and anteriomedial portals with the patient in the sitting position with the knee bent, the target is traditionally the synovial membrane reflections in the intercondylar notch, but these approaches provide only 71-75% accuracy [2,4,6,10-18]. Although the extended leg lateral midpatellar approach has been reported to be highly accurate (93%), there are certain patients where the midpatellar approach may be impractical [2]. A knee injection technique that permits the patient to remain in the sitting position with a bent knee, but provides similar levels of accuracy and outcome as the lateral midpatellar portal would be of clinical utility [2,3,9].

We hypothesized that a modified anteriolateral portal where the synovial membrane of the medial femoral condyle is the needle target would be highly accurate and provide equivalent clinical outcomes as the standard lateral midpatellar approach for intraarticular injection of the knee.

## Methods

### Subjects

This project was in compliance with the Helsinki Declaration, approved by the institutional review board (IRB), and is registered at ClinicalTrials.gov (Clinical Trial Identifier NCT00651625). Inclusion criteria included: 1) osteoarthritis of the knee; 2) Brandt grades 1-3 osteoarthritis as diagnosed by radiographs, 2) persistent pain in the involved joint, 3) significant pain in the involved joint by 10 cm Visual Analogue Pain Sale (VAS) where VAS ≥ 5 cm, 4) failure of exercise and oral analgesics, and 5) the recommendation from the physician for an intraarticular injection [9,19] (6,50). Exclusion criteria included 1) Brandt grade 4 osteoarthritis, 2) hemorrhagic diathesis, 3) the use of warfarin or antiplatelet therapy, or 4) the presence of infection (6). A total of 83 knees with osteoarthritis were randomized between the lateral midpatellar portal (40 knees) and the modified anteriolateral portal (43 knees) (% difference: +8%; 95% CI: -8% to +9%, p = 0.50). Subject age (midpatellar: 61 ± 10 yr; anteriolateral: 59 ± 14 yr; % difference: -3%; 95% CI: -12% to +6%, p = 0.47), female gender (midpatellar: 80%; anteriolateral: 88%; % difference: +13%; 95% CI: -10% to +31%; p = 0.47), subjects who completed study (100% for both, p = 0.5), and pre-procedure baseline pain (10 cm VAS; midpatellar: 8.1 ± 2.0 cm; anteriolateral: 7.6 ± 2.2 cm; % difference: -6%; 95% CI: -18% to +5%; p = 0.28) were similar between the two treatment groups. Outcomes included procedural pain and injection pain, significant pain (VAS ≥ 5 cm), VAS pain scores at outcome (2 weeks and 6 months), mean change in VAS pain scores at outcome, responders (asymptomatic joints at outcome defined by VAS < 2 cm), non-responders (symptomatic joints at outcome defined by VAS ≥ 2 cm), duration of therapeutic effect (months), and time to reinjection or referral to surgery (months). Cost-effectiveness was not calculated because this is highly dependent on reimbursement that varies widely from country to country [20].

### Injection Technique

**Figure 1:**
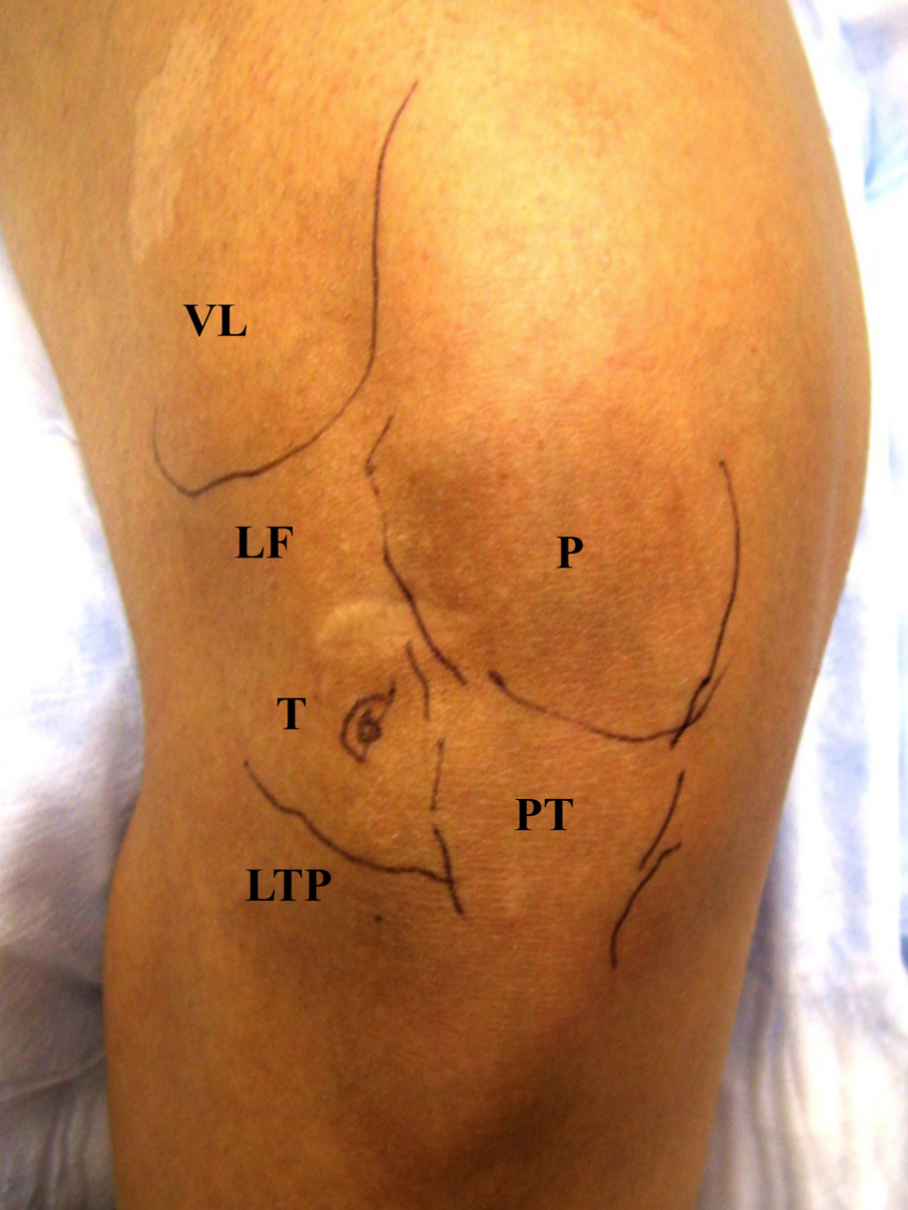
**Palpation-Guided Anatomic Markers**. Anatomic landmarks are first identified by palpation and marked with ink prior to the procedure for both palpation-guided procedures. **VL **(vastus lateralis), **LF **(lateral femoral condyle), **LTP **(lateral tibial plateau), **PT **(patellar tendon), **P **(patella), **T **(target for anteriolateral bent knee approach).

**Figure 2:**
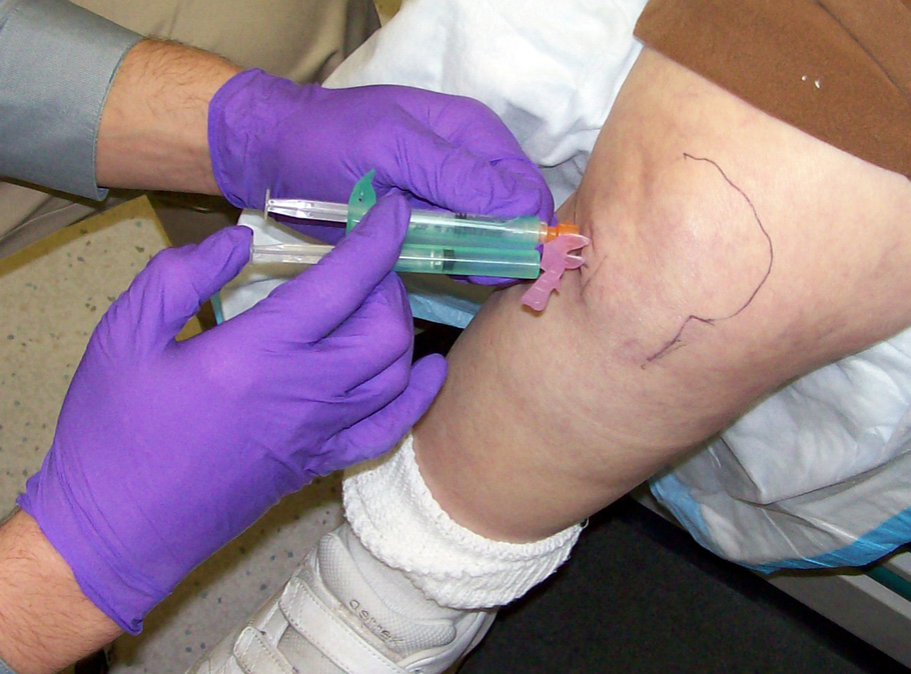
**Introduction of the Needle from the Anteriolateral Portal**. The reciprocating procedure device (RPD) control syringe is used with two hands to carefully introduce the needle and administer lidocaine. Depressing one plunger causes the RPD control syringe to aspirate and depressing the other causes the device to aspirate. If no fluid is obtained the needle is advanced to the medium femoral condyle.

**Figure 3:**
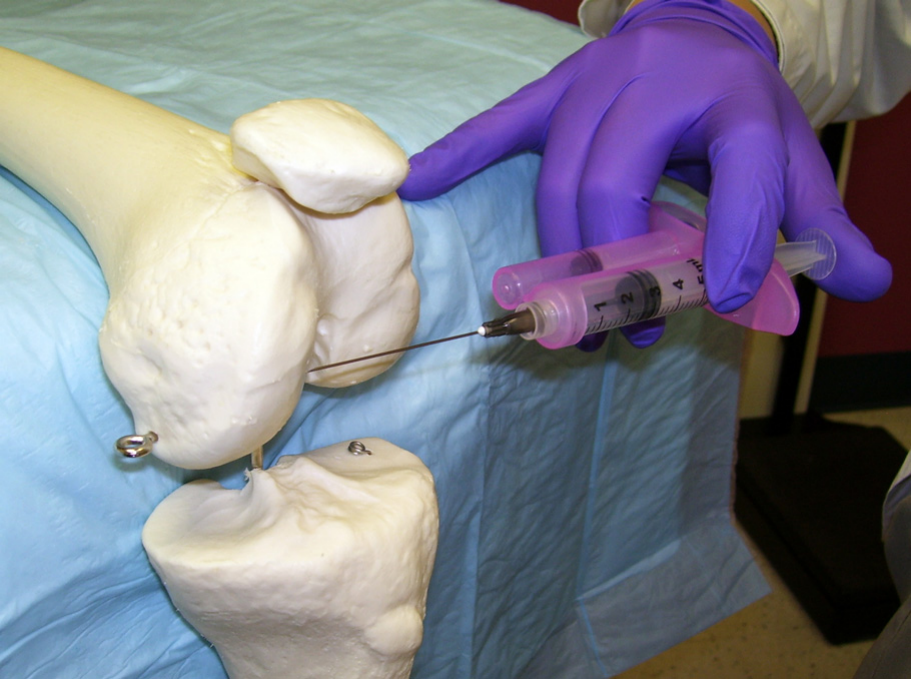
**Modified Anteriolateral Portal**. With the modified anteriolateral portal and the knee in the bent position, the needle is directed from the anteriolateral portal, under the patellar tendon, to the synovial surfaces of the medial femoral condyle rather than the intercondylar notch.

**Figure 4:**
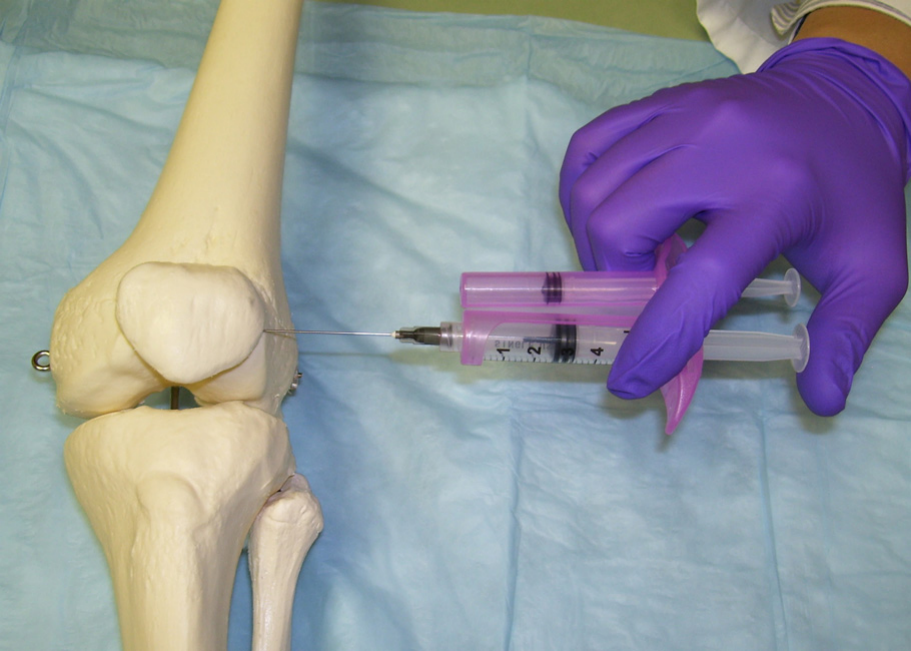
**Lateral Midpatellar Portal**. With the lateral midpatellar portal and the knee is almost fully extended, the needle is directed from the lateral midpatellar position into the patellofemoral joint space.

The one-needle two-syringe technique was used where 1) one needle is used for anesthesia, arthrocentesis, hydrodissection, and intraarticular injection; 2) the first syringe is used to anesthetize, aspirate effusion, and hydrodissect and dilate the joint space, and 3) the second syringe is used to inject the intraarticular therapy [9]. For the modified anteriolateral portal, relevant anatomic landmarks (patella, patellar tendon, lateral tibial plateau, lateral femoral condyle, and medical femoral condyle were palpated and marked with ink (Figure 1 and 2). The anteriolateral portal was defined by the adjoining structures of inferiolateral border of the patellar, the patellar tendon, and the lateral tibial plateau. Rather than directing the needle to the traditional anteriolateral target, the intercondylar notch, the modified anteriolateral approach targets the synovial membrane of the medial femoral condyle (Figure 3) [2,9,21]. This requires directing the needle from the anteriolateral portal under the patellar tendon through the anterior fat pad, until the needle tip directly penetrates the synovial membrane and the bevel engages the medial femoral condyle (Figure 3) [9]. The lateral midpatellar injection was performed with the knee almost fully extended (in 10 to 15 degrees of flexion), and the needle was directed from the lateral surface of the knee to the middle of the patellofemoral joint (Figure 4) [2].

A 21 gauge 2.0 inch (5.1-cm) needle (305783-21 g BD Needle, BD, 1 Becton Drive, Franklin Lakes, NJ 07417, website: http://www.bd.com) was mounted on a 3 ml mechanical syringe, the reciprocating procedure device (RPD syringe) (AVANCA Medical Devices, Inc, Albuquerque, New Mexico, USA. website: http://www.AVANCAMedical.com). For very large individuals a 3.0 inch (7.7 cm) 21 gauge needle was used instead. The RPD mechanical syringe is formed around the core of a conventional syringe barrel and plunger, but has a parallel injection plunger and an accessory barrel to control the motion of the injection plunger (Figure 2, 3, and 4). The two plungers are mechanically linked by a pulley in an opposing fashion, resulting in a set of reciprocating plungers. Thus, when the aspiration plunger is depressed with thumb, the mechanical syringe aspirates, and when the injection plunger is depressed with the thumb, the syringe injects. This permits the index and middle fingers to remain in one position during both aspiration and injection, while the thumb only needs to move in a horizontal plane to the alternative plunger in order to change the direction of aspiration or injection. Mechanical syringes permit easy detection of small amounts of synovial fluid that flash back into the barrel confirming true intraarticular positioning [9,22-25]. Prior to the procedure the mechanical syringe was filled with 1% lidocaine (3 ml for the knee) (Xylocaine^® ^1%, AstraZeneca Pharmaceuticals LP, 1800 Concord Pike, P.O. Box 15437, Wilmington, DE 19850-5437). While alternatively aspirating and injection, the needle tip was directed from the anteriolateral portal across the knee towards the medial femoral condyle until the needle tip penetrated the synovial membrane and the needle encountered firm resistance to further advance (Figure 2 and 3).

After the needle was advanced to the surface of the medial femoral condyle, the lidocaine was injected intraarticularly to hydrodissect and lift the synovial membrane over the needle bevel, and using one hand to hold the mechanical syringe and the other hand the needle hub, the mechanical syringe was rotated off the intraarticular needle, and a 3 ml conventional syringe prefilled with 80 mg triamcinolone acetonide suspension (Kenalog^® ^40, Westwood-Squibb Pharmaceuticals, Inc (Bristol-Myers Squibb), 345 Park Ave, New York, NY 10154-0004, USA) was rotated onto the intraarticular needle, and the treatment was injected. The needle was extracted, and firm pressure applied to the puncture site.

### Determination of Intraarticular Injection Accuracy

**Figure 5:**
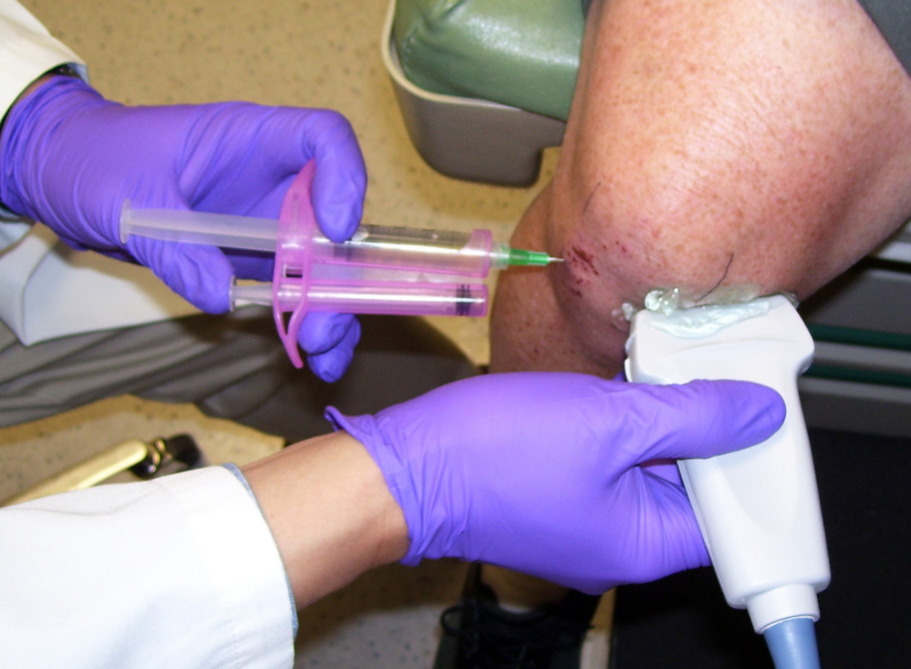
**Placement of Ultrasound Probe**. After introduction of the needle, the ultrasound probe is placed over the anteriomedial portal so that the ultrasound beam is at right angles to the needle shaft optimizing visualization of the location of the needle tip engaged to the medial femoral condyle.

**Figure 6:**
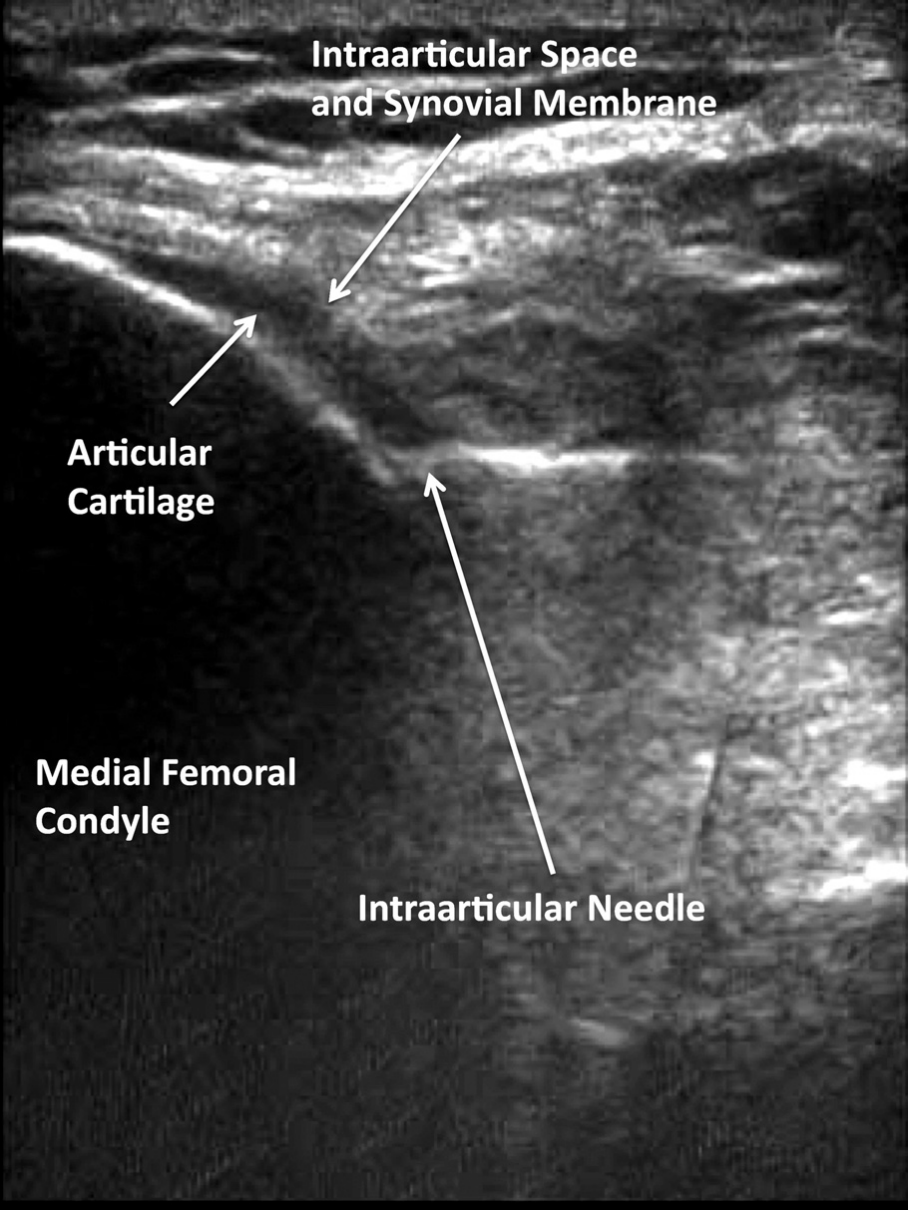
**Sonographic Visualization of Needle Placement**. The needle is advanced until the needle tip palpably engages the medial femoral condyle.

To determine the intraarticular injection accuracy of the modified anteriolateral bent knee portal, 76 consecutive osteoarthritic knees fulfilling the above criteria were injected using the bent knee modified anteriolateral portal, and needle positioning and fluid flow determined by sonography. To determine accuracy of needle placement with sonography, the observer placed the long axis of the ultrasound transducer over the anteriomedial portion of the knee, such that the transducer was parallel to the needle shaft and thus the ultrasound beam vector would best approximate 90 degrees to the long axis of the needle, providing optimal reflected ultrasound signal and excellent visualization of the needle (Figure 5 and 6) [9]. A portable ultrasound unit with a 10-5 MHz 38 mm broadband liner array transducer (Sonosite M-Turbo, SonoSite, Inc. 21919 30th Drive SE, Bothell, WA 98021) was used to sonographically determine the location of the medial femoral condyle, the position of the needle tip, intraarticular and extraarticular fluid flow, and dilation of the intraarticular space.

**Figure 7:**
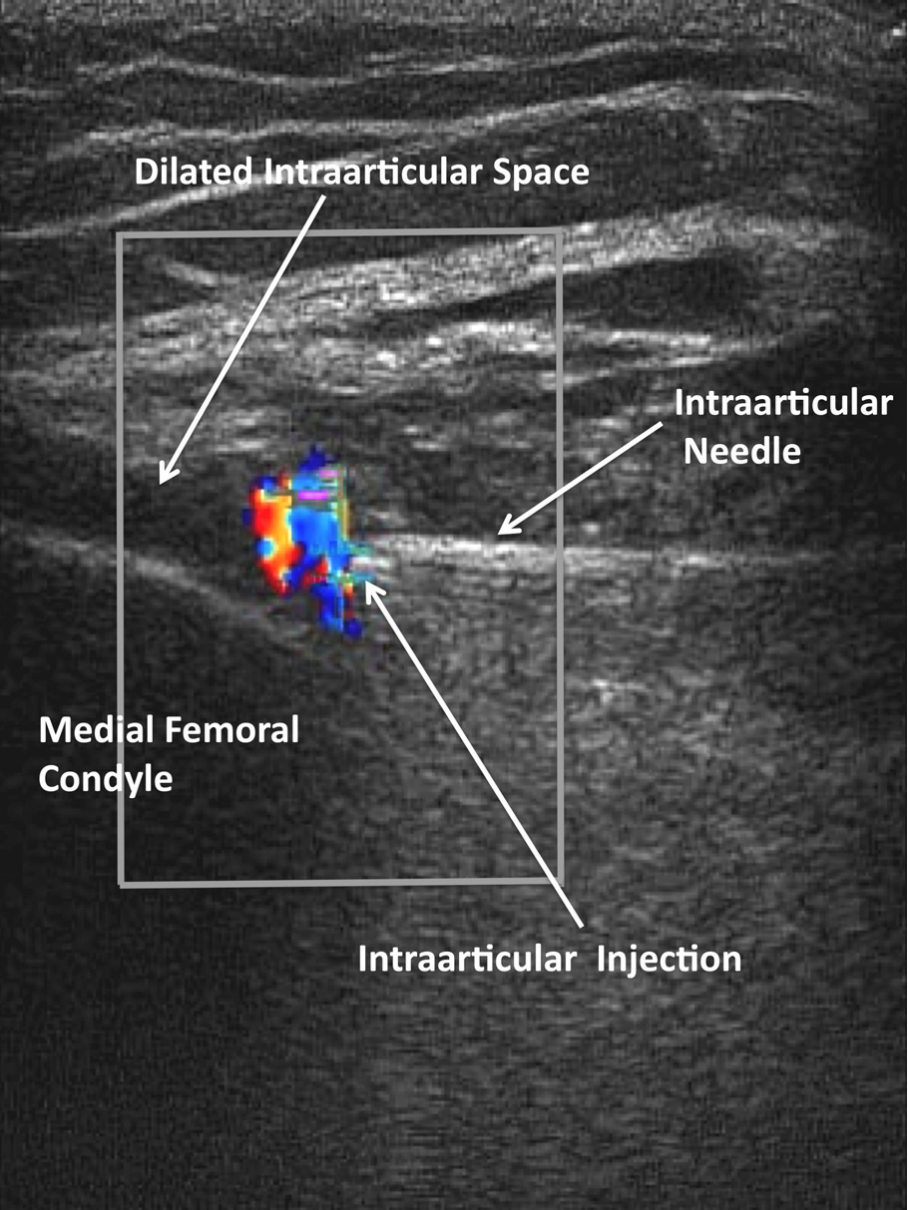
**Sonographic Visualization of Direct Intraarticular Injection**. After the needle tip is advanced until it palpably engages the medial femoral condyle, lidocaine is injected showing fluid movement into the intraarticular space and dilation of the intraarticular space. Here intraarticular movement of fluid is demonstrated by color Doppler at the needle tip with simultaneous dilation of the intraarticular space.

Two positions of the needle tip were tested: 1) when the needle palpably engaged the medial femoral condyle, and 2) when the needle did not palpably engage the medial fem femoral condyle. True intraarticular positioning was established by demonstrating the sonographic characteristics of the needle tip: 1) anatomic positioning of the needle tip at the interface of synovial membrane and cartilage (Figure 3 and 6), 2) demonstration of the free flow of fluid from the needle tip into the intraarticular space and not into the anterior fat pad (Figure 7), and 3) demonstration of dilation of the intraarticular space with the injected fluid [9,14,26-29] (Figure 7). Extraarticular injection was determined by observing 1) fluid movement into the anterior fat pad or retroflow of fluid back along the needle shaft, 2) increased echogenicity in the septa of the anterior fat pad, and 3) lack of fluid movement into and dilation of the intraarticular space [9].

### Statistical Analysis

Data were entered into Excel (Version 5, Microsoft, Seattle, WA), and analyzed in SAS (SAS/STAT Software, Release 6.11, Cary, NC). Differences in measurement data were determined with the student t-test; categorical data were determined with Fisher's Exact Test, while differences between multiple parametric data sets were determined with Fishers Least Significant Difference Method.

## Results

Table 1 demonstrates the similarities in short-term outcome between the lateral midpatellar and modified anteriolateral portals for injecting the knee. As can be, seen there were no differences in pre-procedure pain (p = 0.28), needle introduction pain (p = 0.77), significant needle introduction pain (p = 0.74), injection pain (p = 0.92), or significant injection pain between the two portals (p = 0.90). Table 2 demonstrates the intermediate (2 week) and long-term outcome (6 months). As can be seen, there were no differences in pain at 2 weeks (p = 0.11), reduction in pain from baseline (p = 0.13), responder rate (p = 0.33), and non-responder rate (p = 0.33). There were also no differences in pain at 6 months (p = 0.89), duration of therapeutic effect (p = 0.69), or time to next procedure (p = 0.71).

**Table 1:** Short-Term Outcomes for Intraarticular Injection of the Knee

	Lateral Midpatellar Portal	Modified Anteriolateral Portal			
**Number of Subjects**	**40**	**43**	**Percent Difference**	**95% Confidence Interval**	**P Value**

**Pre-Procedure Baseline Pain (VAS)**	8.1 ± 2.0 cm	7.6 ± 2.2 cm	-6%	-18% to +5%	0.28

**Needle Introduction Pain (VAS)**	4.6 ± 3.1 cm	4.8 ± 3.2 cm	+4%	-26% to +34%	0.77

**Significant Needle Introduction Pain (VAS ≥ 5 cm)**	48% (19/40)	44% (19/43)	-6%	-49% to +37%	0.74

**Injection Pain (VAS)**	2.8 ± 2.8 cm	2.9 ± 3.4 cm	+4%	-45% to +53%	0.92

**Significant Injection Pain (VAS ≥ 5 cm)**	23% (9/40)	21% (9/43)	+2%	-71% to +86%	0.90

**Table 2:** Intermediate-Term and Long-Term Outcomes of IntraarticularInjection of the Knee

	Lateral Midpatellar Portal	Modified Anteriolateral Portal			
**Number of Subjects**	**40**	**43**	**Percent Difference**	**95% Confidence Interval**	**P Value**

**Pain at Outcome (2 weeks) (VAS)**	2.6 ± 2.8 cm	1.7 ± 2.3 cm	-35%	-78% to +9%	0.11

**Reduction in Pain from Baseline (VAS)**	4.4 ± 3.4 cm	5.5 ± 3.1 cm	+25%	-7% to +57%	0.13

**Responders (VAS < 2 cm)**	45% (18/40)	56% (24/43)	+24	-23% to +68%	0.33

**Non-Responders (VAS ≥ 2 cm)**	55% (22/40)	44% (19/43)	-20%	-56% to +19%	0.33

**Pain at Outcome (6 months) (VAS)**	4.9 ± 3.1 cm	4.8 ± 3.2 cm	-2%	-30% to +26%	0.89

**Duration of Therapeutic Effect**	3.9 ± 2.4 months	4.1 ± 2.2 months	+5%	-21% to +31%	0.69

**Time to Next Procedure (reinjection or referral to surgery)**	7.3 ± 3.3 months	7.7 ± 3.7 months	+6%	-16% to +27%	0.71

Accuracy of intraarticular injection using the bent knee modified anteriolateral portal was highly dependent on needle tip positioning. When the needle tip was not positioned to directly and palpably engage the medial femoral condyle, true intra-articular injection was only observed in 22% (17/76) of the test injections with the bulk of the injection being deposited in the anterior fat pad. When the needle was positioned so that it palpably engaged the medial femoral condyle, after hydrodissection with lidocaine, accurate intraarticular injection was demonstrated in 97% (74/76) of knees, resulting in a +332% (95% CI: +289% to +372%) increase in intraarticular injection success, significantly better than when the needle was not positioned to engage the condyle (p < 0.001). Although the 2 inch (5.1 cm) needle was used in the majority of subjects, the needle was hubbed or nearly hubbed against the skin in larger individuals in order to palpably engage the medial femoral condyle, thus, the use of the 3 inch (7.7 cm) needle or longer provided more leeway in large or obese individuals.

## Discussion

The non-effusive or "dry joint" typical of osteoarthritis of the knee is particularly challenging to the sports medicine proceduralist, and selection of anatomic approach is of critical importance to ensure accuracy [2,16,17,21]. Several methods have been proposed to increase the accuracy of intraarticular placement of the needle in the absence of an effusion. These include careful selection of the anatomic portal, preinjection of air, saline or lidocaine to hydrodissect and dilate the intraarticular space, aspiration of droplets of synovial fluid or moisture into the barrel of the syringe, minimal retraction of the needle tip after palpable engagement of an articular cartilage or bone surface, use of highly controlled mechanical syringes, the one-needle two syringe technique, ultrasound guidance, or fluoroscopic injection of contrast material [2,4,6,9-18].

Jones et al demonstrated that only 66% of palpation-guided knee injections were truly intraarticular, while Bliddal demonstrated that the superiolateral approach was 91% accurate [11,13]. In a study with 11 subjects in each arm, Toda et al demonstrated a that the modified Waddell approach (an anteriomedial approach with manipulative ankle traction at 30 degrees of knee flexion) was 100% accurate, bent knee anteriomedial approach was 55% accurate, and the anteriolateral approach was 55% accurate [30]. Lopes et al reported 100% accuracy in knee injection rates, but these were in patients with rheumatoid arthritis where the target - the synovial mass (both tissue and effusions) - is much larger than in osteoarthritis [31]. In contrast, Cunnington et al reported only 66.3% accuracy in inflammatory arthritis with palpation-guided methods [32]. In a cadaver study Esenyel et al have demonstrated a 56% to 85% intraarticular injection accuracy depending on anatomic approach with the anteriolateral approach being the most accurate and the medial midpatellar portal being the least accurate [21]. Wind et al demonstrated that the superiomedial and superiolateral injections into the knee were the most accurate, while the lateral joint line injection was the most inaccurate [33]. Jackson et al demonstrated that lateral midpatellar approach was the most accurate (93% accuracy) while the anteriomedial and anteriolateral portals were less accurate (71% and 75%, respectively) [2]. The target for the anteriolateral and anteriomedial portals is generally the intercondylar notch, which contains the cruciate ligaments and associated synovial membrane reflections [2,21].

For those apprehensive individuals who involuntarily and forcefully contract the quadriceps muscles during a procedure, the elderly, individuals with knee contractures, the obese, or wheelchair-bound individuals, the lateral midpatellar approach can be difficult and/or inconvenient in these individuals. In addition since less subcutaneous fat is transversed by the needle in the lateral midpatellar portal, local complications of injections are more easily observed, including visible ecchymosis, hematoma, and cutaneous atrophy or foreign body granuloma formation at the puncture site caused by reflux of corticosteroid or hyaluron back through the needle tract [2,34-39]. Thus, refinement of a method of injecting the knee that permits the patient to remain in the sitting position with a bent knee and does not require forcing the needle into the constrained anatomy of the patellofemoral joint, but provides similar levels of accuracy as the lateral midpatellar portal would be of clinical utility in certain individuals [2,21].

Because of the associated cruciate ligaments and reflections of the synovial membrane, the intercondylar notch presents a narrow anatomic target where the synovial space and the araeolar synovial tissue cannot be easily distinguished while advancing the needle tip, making true anatomic injection difficult, resulting in only a 71% to 75% accuracy rate [2]. We hypothesized that a modified anteriolateral portal where the synovial membrane overlying the medial femoral condyle is the needle target (Figure 3) would be accurate and provide equivalent clinical outcomes as the standard lateral midpatellar approach (Figure 4). The modified anteriomedial bent knee portal proved highly accurate (97% intraarticular accuracy) when the needle tip could be felt engaging the medial femoral condyle, which compares favorably with the 93% accuracy with the lateral midpatellar approach [2]. Moreover, the modified bent knee anteriolateral portal provided equivalent clinical outcomes to the standard lateral midpatellar approach (Table 1 and 2).

There are limitations to this study. First, this study employed the one-needle two-syringe technique where a first syringe is used to anesthetize, aspirate, hydrodissect, and dilate the joint space. This technique may improve intraarticular accuracy and outcomes independent of anatomic portal because this maneuver in fact hydrodissects and lifts the synovial membrane over the needle bevel increasing true intraarticular injection accuracy [9]. Similarly, the one-needle two-syringe technique permits aspiration of synovial fluid prior to injection further confirming intraarticular injection accuracy, decompressing the joint, and improving outcomes as opposed to direct injection without aspiration that does not [10,13,40,41].

The present study used 1% lidocaine for local anesthesia and hydrodissection. Although the local anesthetic bupivacaine has resulted in chondrolysis and malpractice actions, short-term exposure to low levels of lidocaine as used in this study have not been demonstrated to be chondrotoxic in vivo; furthermore, cases of chondrolysis with lidocaine have not been reported in large case series [10,23,42-45]. Lidocaine and chlorhexidine have the additional benefit in that they are potent bactericidal agents and may contribute to the low incidence of joint infections and lack of chondrolysis associated with intraarticular injections [23,36,46-49].

Another aspect of the modified anteriolateral injection technique is that a 2 to 3 inch (5.1 to 7.7 cm) needle (depending on knee subcutaneous and skeletal dimensions) is necessary for this technique to be predictably accurate so that the needle tip can effectively access the joint space and palpably engage the medial femoral condyle. Since the 2 inch needle was hubbed or nearly hubbed in many individuals in order to palpably engage the medial femoral condyle, the use of the more common 1.5 inch (3.8 cm) needle would likely to substantially reduce the accuracy rates because the needle is too short to reach the synovial space overlying the medial femoral condyle and would only be useful in smaller individuals. Also the use of the mechanical syringe rather than a conventional syringe may have contributed to improved intraarticular accuracy independent of portal as mechanical syringes have been shown to be better controlled and more accurate than a conventional syringe, and reduce procedural trauma, accentuate synovial fluid detection and removal, and improve intraarticular injection outcomes with injection of local anesthetics, hyaluron, and corticosteroid [9,23-25,50-55].

## Conclusions

In summary, the palpation-guided bent knee modified anteriolateral portal for intraarticular injection of the knee where the synovial space overlying the medial femoral condyle is the needle target is a highly accurate alternative method to the lateral midpatellar portal and provides high levels of accuracy with minimal pain while maintaining excellent injection outcomes.

## Competing interests

CEC-C, NRC-C, SLD, and ADB have nothing to disclose. PAB is an employee of New York University, and is a consultant to Smith & Nephew, Inc., and Avanca Medical Devices, Inc. WLS is funded by Research Grant RO1 HLO77422-01-A3 from the US National Institutes of Health and by a Grant from the US Public Health Service. WLS also is an expert consultant for Intelligence Management Solutions, Inc., Ferring Pharmaceuticals, Inc., Avanca Medical Devices, Inc; Avasca, Inc., Becton-Dickinson, Inc., Surgin, Inc., and MediTech Duopross, Inc. WLS holds stock in Apple Inc, Celgene Corp, Inc, Avasca, Inc., Avanca, Inc, Sun Microsystems, Inc, Symantec Corp, and Java, Inc. In 2009 Abbott Vascular, Inc. acquired 4 patents invented by WLS, but these patents are not relevant to the present research.

## Authors' contributions

CEC-C performed literatures searches, wrote part of the text, and contributed to the analysis and discussion. NRC-C and SLD performed many of the ultrasound studies and injection procedures, WLS, PAB, and ADB conceived the study, supervised the studies, analyzed data, and drafted the manuscript. All authors read and approved the final manuscript.
